# Characterisation of the transcriptome and proteome of SARS-CoV-2 reveals a cell passage induced in-frame deletion of the furin-like cleavage site from the spike glycoprotein

**DOI:** 10.1186/s13073-020-00763-0

**Published:** 2020-07-28

**Authors:** Andrew D. Davidson, Maia Kavanagh Williamson, Sebastian Lewis, Deborah Shoemark, Miles W. Carroll, Kate J. Heesom, Maria Zambon, Joanna Ellis, Philip A. Lewis, Julian A. Hiscox, David A. Matthews

**Affiliations:** 1 School of Cellular and Molecular Medicine, Faculty of Life Sciences, University Walk, University of Bristol, Bristol, BS8 1TD UK; 2 School of Biochemistry, Faculty of Life Sciences, University Walk, University of Bristol, Bristol, BS8 1TD UK; 3 Research and Development Institute, National Infection Service, Public Health, England, Porton Down, Wiltshire, UK; 4 National Institute Health Research, Health Protection Research Unit in Emerging and Zoonotic Infections, Liverpool, UK; 5 Proteomics Facility Faculty of Life Sciences, University Walk, University of Bristol, Bristol, BS8 1TD UK; 6 Virus Reference Department, Public Health England (Colindale), London, UK; 7 Institute of Infection, Veterinary and Ecological Sciences, University of Liverpool, Liverpool, UK; 8 Liverpool Health Partners, Liverpool, UK

## Abstract

**Background:**

SARS-CoV-2 is a recently emerged respiratory pathogen that has significantly impacted global human health. We wanted to rapidly characterise the transcriptomic, proteomic and phosphoproteomic landscape of this novel coronavirus to provide a fundamental description of the virus’s genomic and proteomic potential.

**Methods:**

We used direct RNA sequencing to determine the transcriptome of SARS-CoV-2 grown in Vero E6 cells which is widely used to propagate the novel coronavirus. The viral transcriptome was analysed using a recently developed ORF-centric pipeline. Allied to this, we used tandem mass spectrometry to investigate the proteome and phosphoproteome of the same virally infected cells.

**Results:**

Our integrated analysis revealed that the viral transcripts (i.e. subgenomic mRNAs) generally fitted the expected transcription model for coronaviruses. Importantly, a 24 nt in-frame deletion was detected in over half of the subgenomic mRNAs encoding the spike (S) glycoprotein and was predicted to remove a proposed furin cleavage site from the S glycoprotein. Tandem mass spectrometry identified over 500 viral peptides and 44 phosphopeptides in virus-infected cells, covering almost all proteins predicted to be encoded by the SARS-CoV-2 genome, including peptides unique to the deleted variant of the S glycoprotein.

**Conclusions:**

Detection of an apparently viable deletion in the furin cleavage site of the S glycoprotein, a leading vaccine target, shows that this and other regions of SARS-CoV-2 proteins may readily mutate. The furin site directs cleavage of the S glycoprotein into functional subunits during virus entry or exit and likely contributes strongly to the pathogenesis and zoonosis of this virus. Our data emphasises that the viral genome sequence should be carefully monitored during the growth of viral stocks for research, animal challenge models and, potentially, in clinical samples. Such variations may result in different levels of virulence, morbidity and mortality.

## Background

Since the emergence of severe acute respiratory syndrome coronavirus-2 (SARS-CoV-2) as a human pathogen at the end of 2019, the virus has spread globally, causing almost 11.1 million confirmed cases of COVID-19 and over half a million deaths as of the 7 July 2020 [1]. Although vaccines are under rapid development to prevent SARS-CoV-2 infection, little is known of either the immune correlates of protection or the ability of the virus to avoid the host immune response through mutation and recombination [2].

**Fig. 1:**
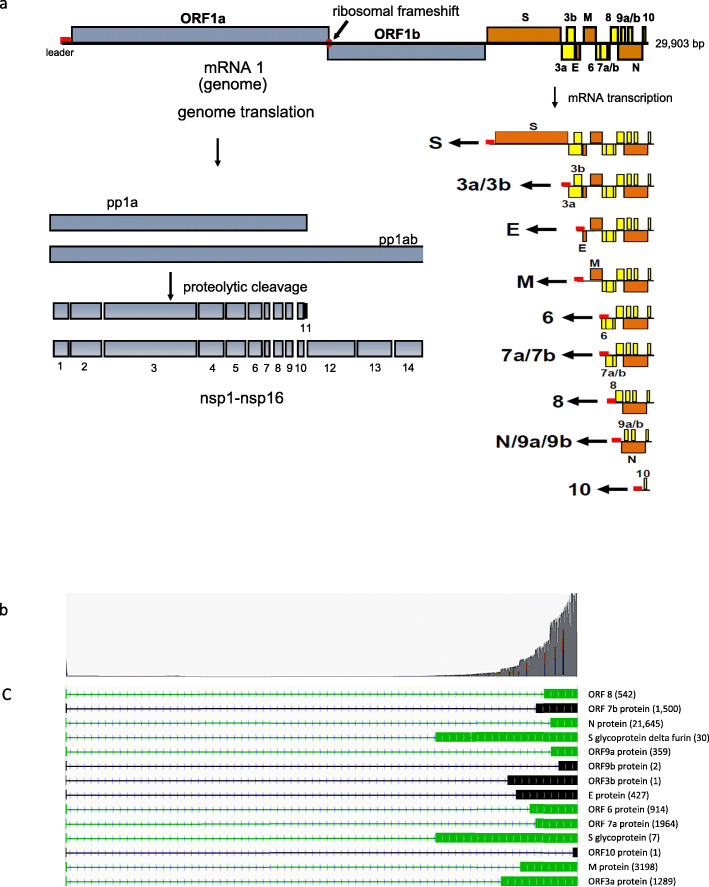
Overview of nanopore inferred transcriptome. **a** The classical transcription map of coronaviruses adapted for SARS-CoV-2. The genome is itself an mRNA which when translated gives rise to polyproteins pp1a and, upon a ribosomal frameshift, pp1ab. These polyproteins are proteolytically processed down to a range of non-structural proteins termed nsp1–16, some of which will form the viral replication-transcription complex (RTC). The RTC then generates subgenomic mRNA which canonically contains a sequence present at the 5′ end of the viral genome known as the leader sequence. The 3′-end of the leader sequence has a motif, the transcription regulatory sequence (TRS), and there are similar sequences which precede each of the functional ORFs downstream of the replicase gene (pp1ab). This TRS in the leader associates with one of the TRS regions present adjacent to each of the other functional ORFS and this mediates discontinuous transcription between the two during minus-strand RNA synthesis. These minus-strand RNA molecules are used as templates to generate positive sense mRNA, and in this manner, the remaining ORFs on the viral genome are placed 5′ most on the resulting subgenomic mRNAs and are subsequently translated. Orange boxes represent structural proteins and yellow boxes represent accessory proteins. **b** The total read depth across the viral genome for all reads; the maximum read depth was 511,129. **c** The structure of only the dominant transcript that codes for each of the identified ORFS. Only transcripts that start inside the leader TRS sequence are considered here. The rectangles represent mapped nucleotides and the arrowed lines represent regions of the genome that are not transcribed during the generation of mRNAs. To the right is noted the 5′ most ORF encoded in the transcript; in parenthesis we note how many individual transcripts were observed. Transcrits coding for proteins we subsequently detected by MS/MS are coloured in green

The genome sequence of SARS-CoV-2 was rapidly determined and revealed the virus is most closely related to bat coronavirus RaTG13 and clusters phylogenetically with SARS-CoV, which emerged in 2002, in the genus *Betacoronavirus* of the family *Coronaviridae* [3, 4]. Based on homology to other known coronaviruses [3, 4], the genome sequence was used for viral transcript prediction and the annotation of ORFs. Coronaviruses use a complex strategy to express their genetic information [5, 6], involving a process of discontinuous transcription during minus-strand RNA synthesis that is regulated by defined transcription regulatory sequences (TRS) [7]. Similar, to other coronaviruses, the SARS-CoV-2 29.9 kB RNA genome contains two large ORFs, ORF1a and ORF1ab, predicted to be initially translated into the polyproteins, pp1a and pp1ab that arises by programmed (− 1) ribosomal frameshifting (Fig. 1a). The polyproteins are post-translationally processed by viral encoded proteases to produce 16 proteins that are conserved between coronaviruses and proposed to function in the synthesis of viral RNA and immune evasion [8]. During viral genome replication a set of “nested” subgenomic mRNAs are produced that are predicted to encode the structural proteins spike (S), envelope (E), membrane (M) and nucleocapsid (N) and at least nine small accessory proteins, some of which are unique to SARS-CoV-2 [3, 4]. The subgenomic mRNAs have a common 5′ leader sequence and are 3′ co-terminal with a polyA tail derived from the viral genome.

Recently, the SARS-CoV-2 transcriptome has been examined using direct RNAseq (dRNAseq) [9, 10]. Furthermore, a recent proteomic study showed that a number of the predicted viral proteins are produced in infected cells, but not all of the predicted viral proteins were detected [11]. We pioneered the use of combining transcriptomics and proteomics to study viral infections in vitro and in vivo, initially using the well characterised respiratory virus, adenovirus but also respiratory syncytial virus and highly pathogenic zoonotic viruses such as Hendra virus [12–14]. More recently, we have focussed on the latest technologies to study the transcriptome of viruses, including direct RNA sequencing on nanopore devices using long read length sequencing. In particular, we developed an ORF-centric pipeline to analyse the very large amounts of transcriptomic data generated by dRNAseq [15]. This pipeline was first used on data generated from adenovirus-infected cells. The approach rapidly reproduced transcript maps that correlated highly with the previously described transcriptome maps of adenovirus, which have been carefully curated over several decades.

The SARS-CoV-2 subgenomic mRNAs encoding the structural proteins and their expression profiles are of particular interest as the encoded proteins are likely targets of a protective immune response. The coronavirus S glycoprotein is present as a homotrimer, protruding from the surface of the virion. It is a key determinant of viral tropism and the major antigenic target [16, 17]. The S glycoprotein is a class I fusion protein comprised of two domains, S1 and S2, responsible for receptor binding and the fusion of viral and cellular membranes respectively [18, 19]. The S glycoprotein of coronaviruses is primed for cell entry by host cell proteases that cleave the protein at one or more positions. Cleavage of a “S1/S2” site at the boundary of the S1 and S2 domains occurs for some coronaviruses whilst cleavage at an “S2” site is common to all coronaviruses and results in activation of a highly conserved fusion peptide immediately downstream of the S2 site [20–22]. Although the SARS-CoV-2 S glycoprotein shares 97% identity with that of bat coronavirus RaTG13 [4], an important difference is the presence of a four amino acid insertion (_681_PRRA_684_) at the S1/S2 junction that introduces a potential furin-like cleavage site [23, 24]. The furin cleavage site is also not found in other “lineage B” betacoronaviruses including SARS-CoV, but has been detected in betacoronaviruses in other lineages, suggesting that this region has arisen during natural evolution [25].

Here, we used a combined transcriptomics and proteomics approach to produce a correlated transcriptome and proteome map of SARS-CoV-2 in the African Green monkey kidney cell line Vero E6. This cell line is routinely used to propagate viruses from clinical samples as well as to generate stocks of virus for academic research, drug susceptibility testing and vaccine challenge studies. Our results show a close correlation between the predicted viral transcriptome and proteome maps as well as novel transcripts that encoded for proteins detected by tandem mass spectrometry. Finally, evidence was gathered for an in-frame deletion inside the S glycoprotein that removed the proposed furin cleavage site.

## Methods

### Virus growth and assay

Vero E6 (ATCC® CRL 1586™) cells were cultured at 37 °C in Dulbecco’s modified Eagle’s medium (DMEM, Gibco™, ThermoFisher) supplemented with 10% foetal bovine serum (FBS), penicillin (100 units/ml) and streptomycin (100 μg/ml). All work with infectious SARS-CoV-2 strain England/2/2020 (VE6-T), isolated by Public Health England (PHE), was done inside a class III microbiological safety cabinet in a containment level 3 facility at the University of Bristol. A SARS-CoV-2 stock was produced by infecting Vero E6 cells at a multiplicity of infection (MOI) of 0.01 and incubating the cells for 72 h. The culture supernatant was clarified by centrifugation and stored in aliquots at − 80 °C. The titre of the stock was determined by preparing 10-fold serial dilutions in Eagle’s minimal essential medium (MEM; Gibco™, ThermoFisher) supplemented with 2% FBS. Aliquots of each dilution were added to 1 × 10^4^ Vero E6 cells in the same medium in each of 12 wells of a 96-well plate. Plates were incubated at 37 °C for 5–7 days and then examined for cytopathic effect. The TCID_50_ was calculated according to the method of Reed and Muench [26].

### RNA extraction for direct RNA sequencing

Extraction and sequencing was done as previously described [15]. Briefly, total RNA was extracted using TRIzol™ reagent (#15596026, Ambion) and the RNA extracted as per the manufacturer’s recommendations but with 3 × 70% ethanol washes. After resuspension in RNAse-free water, the RNA was polyA enriched and sequenced immediately using SQK-RNA002 kits and MIN106D R9 version flow cells (Oxford Nanopore Technologies).

### Data analysis, characterisation of viral transcripts

To cope with the very wide range of transcripts, and to enable grouping of transcripts into classes, our previously described ORF-centric data analysis pipeline was utilised [15]. The transcripts were mapped to the viral genome with minimap2, and the mapping data was used to try to identify transcription regulatory sequences (TRS) and the sites where the transcript meets the polyA tail at the end of the genome. This is complicated by the repetitive nature of the leader and body TRS and thus mapping software struggles to determine where the breakpoint should map. Canonical and non-canonical junction sites were also identified, which the software refers to as splice acceptor/donor sites as it was originally used to describe spliced adenovirus transcripts. This script produces tables indicating where on the genome and how often in the data each junction occurs. Subsequently, nearby events are grouped together for simplicity of analysis. Once this is complete, the software then assigns each transcript to a “transcript group” depending on its pattern of TRS, junction sites and poly A locations and counts how many transcripts belong to each transcript group. Nanopolish [27] was used to determine the polyA length of each sequenced transcript and subsequently a simple average polyA length was calculated for each transcript group alongside the standard deviation.

A second in-house script determined which known features are present in each transcript group and generated pseudo-transcripts based on the viral genome sequence to remove nanopore sequencing errors. The script examined each pseudo-transcript to determine what features it has, using a user-specified list of canonical features or ORFs on the viral genome (Additional file 1). It also produced GFF files that allow the user to visualise only the dominant transcript coding for each ORF as well as GFF files describing the whole range of transcripts coding for any given ORF. In addition, an analysis counting the final number of transcripts belonging to each translated feature was produced. The pipeline generated an ORF-centric view of the viral transcriptome—classifying transcripts according to the encoded viral proteins.

### RNA extraction for sequencing of the suspected deletion region

To prepare intracellular SARS-CoV-2 RNA, total cellular RNA containing SARS-CoV-2 RNA was extracted from the Vero E6 cells used for viral stock production using TRIzol™ Reagent (Invitrogen™, ThermoFisher) following the manufacturer’s instructions. Viral RNA was extracted from cell culture supernatants using a QIAamp Viral RNA Mini Kit (Qiagen) according to the manufacturer’s instructions. Approximately 3 kb RT-PCR products covering the S gene deletion were amplified from the viral RNA using the gene specific primers F9newF and F9newR (5′-TAAGGTTGGTGGTAATTATAATTACCTG-3′ and 5′-AAAATAGTTGGCATCATAAAGTAATGGG-3′) and a SuperScript™ IV One-Step RT-PCR System (Invitrogen™, ThermoFisher). A region spanning the deletion was sequenced using primers Wu_24_L and Wu_24_R (5′-TTGAACTTCTACATGCACCAGC-3′ and 5′-CCAGAAGTGATTGTACCCGC-3′).

### Total proteome analysis

Protein lysates were prepared from the Vero E6 cells used for viral stock production. The cells were harvested in 4X Laemmli buffer (BioRad) and heated to 95 °C for 15 min. A 25-μl aliquot of the sample was separated using SDS-PAGE and the gel lane cut into 20 slices. The slices were reduced (10 mM DTT, 56 °C, 30 min), alkylated (100 mM iodoacetamide, room temperature, 20 min) and digested with trypsin (0.5 μg trypsin per slice, 37 °C, overnight). This whole process was repeated with chymotryptic digestion (0.5 μg chymotrypsin per slice, 25 °C, overnight). The resulting tryptic and chymotryptic peptides were fractionated using an Ultimate 3000 nano-LC system in line with an Orbitrap Fusion Lumos mass spectrometer (Thermo Scientific). In brief, the peptides from each gel slice in 1% (vol/vol) formic acid were injected onto an Acclaim PepMap C18 nano-trap column (Thermo Scientific). After washing with 0.5% (vol/vol) acetonitrile 0.1% (vol/vol) formic acid, peptides were resolved on a 250 mm × 75 μm Acclaim PepMap C18 reverse phase analytical column (Thermo Scientific) over a 150 min organic gradient, using 7 gradient segments (1–3% solvent B over 1 min, 3–15% B over 58 min, 15–32%B over 58 min, 32–40%B over 5 min, 40–90%B over 1 min, held at 90%B for 6 min and then reduced to 1%B over 1 min) with a flow rate of 300 nl min^−1^. Solvent A was 0.1% formic acid and solvent B was aqueous 80% acetonitrile in 0.1% formic acid. Peptides were ionised by nano-electrospray ionisation at 2.2 kV using a stainless-steel emitter with an internal diameter of 30 μm (Thermo Scientific) and a capillary temperature of 250 °C.

All spectra were acquired using an Orbitrap Fusion Lumos mass spectrometer controlled by Xcalibur 3.0 software (Thermo Scientific) and operated in data-dependent acquisition mode. FTMS1 spectra were collected at a resolution of 120,000 over a scan range (m/z) of 375–1550 (for tryptic peptides) or 325–1500 (for chymotryptic peptides), with an automatic gain control (AGC) target of 4E5 and a max injection time of 50 ms. Precursors were filtered according to charge state (to include charge states 2–7), with monoisotopic peak determination set to peptide and using an intensity threshold of 1E3. Previously interrogated precursors were excluded using a dynamic window (40s ± 10 ppm). The MS2 precursors were isolated with a quadrupole isolation window of 0.7 m/z. ITMS2 spectra were collected with an AGC target of 2E4, max injection time of 35 ms and HCD collision energy of 30%.

A targeted analysis was performed to confirm the identification of the S protein deletion specific peptide (QTQTIASQSIIAY) identified by a single peptide spectral match (PSM) in the initial analysis of chymotryptic peptides. However, there were changes to the acquisition workflow. Precursors were filtered according to charge state (to include charge state 2) and previously interrogated precursors were excluded using a dynamic window (2 s ± 10 ppm). A targeted mass was specified with m/z 712.3759 and *z* = 2.

### Phosphoproteome analysis

Six 30 μl aliquots of the infected cell total protein lysate were separated by SDS-PAGE until the dye front had moved approximately 1 cm into the separating gel. Each gel lane was excised as a single slice and subjected to in-gel tryptic digestion as above but using 1.5 μg trypsin per slice. The resulting peptides were subjected to TiO_2_-based phosphopeptide enrichment according to the manufacturer’s instructions (Pierce). The flow-through and washes from the TiO_2_-based enrichment were then subjected to FeNTA-based phosphopeptide enrichment, again according to the manufacturer’s instructions (Pierce). The phospho-enriched samples were evaporated to dryness and then resuspended in 1% formic acid prior to analysis by nano-LC MSMS using an Orbitrap Fusion Lumos mass spectrometer (Thermo Scientific) as above.

### Data analysis

The raw data files were processed using Proteome Discoverer software v2.1 (Thermo Scientific) and searched against the UniProt *Chlorocebus sabaeus* database (downloaded March 2020; 19,525 sequences), an in-house “common contaminants” database and a custom SARS-CoV-2 protein database using the SEQUEST HT algorithm. Peptide precursor mass tolerance was set at 10 ppm, and MS/MS tolerance was set at 0.6 Da. Search criteria included oxidation of methionine (+ 15.995 Da), acetylation of the protein N-terminus (+ 42.011 Da) and methionine loss plus acetylation of the protein N-terminus (− 89.03 Da) as variable modifications and carbamidomethylation of cysteine (+ 57.021 Da) as a fixed modification. For the phosphoproteome analysis, phosphorylation of serine, threonine and tyrosine (+ 79.966 Da) was also included as a variable modification. Searches were performed with full tryptic or chymotryptic digestion and a maximum of 2 missed cleavages were allowed. The reverse database search option was enabled and all data was filtered to satisfy a false discovery rate (FDR) of 5%.

### Data availability

The fastq files and ThermoFisher .raw files are available on zenodo.org under the following doi's: 10.5281/zenodo.3722580 for the fastq data [28], 10.5281/zenodo.3722604 for the phosphoproteomics .RAW files [29], 10.5281/zenodo.3722590 for the total proteome .RAW files (slices 1–10 of 20) [30] and 10.5281/zenodo.3722596 for the total proteome .RAW files (slices 11–20 of 20) [31]. The proteomics data has also been deposited at PRIDE (PXD018241) [32] and the transcriptomics data at the European Nucleotide Archive (PRJEB39337) [33].

## Results

Complete characterisation of a novel and dangerous pathogen at a molecular level is fundamental. Although analysis of the SARS-CoV-2 genome enables high confidence prediction of potential transcripts and ORFS, there is a pressing need to have the predictions confirmed and novel transcripts identified and assessed for biological significance and their role in virulence and pathogenesis. At the same time, there is also a need for independent confirmation that viral transcripts are expressed, which can be achieved by tandem mass spectrometry-based proteomics. In this way, we have provided direct observation of the whole range of viral proteins expressed in SARS-CoV-2-infected cells and helped resolve ambiguities in the transcriptomic data. Finally, the identification of phosphorylation sites provided an invaluable list of potential therapeutic targets based on kinase inhibitors. Using proteomics informed by transcriptomics (PIT) [12] on duplicate infected cells offers a rapid and high resolution approach to these key molecular virology questions.

### Overview of dRNAseq outputs

Long read length sequencing using an Oxford Nanopore MinION on polyA+ RNA from cells infected with SARS-CoV-2 was used to characterise viral RNA species. This technique has recently been used to study herpes viruses, coronaviruses and adenovirus transcriptomes [15, 34, 35]. In total, 1,588,330 sequences were base called and passed QC; of those, 527,401 were mapped to the BetaCoV/England/02/2020 genome using minimap2 (Fig. 1b). In addition, an average polyA tail length for each transcript was generated using nanopolish [27] and this information was added to the analysis pipeline. A cutoff of a 20 nt minimum polyA length was employed and 386,903 transcripts passed this test. Finally, the data from the analysis pipeline was tabulated describing the structure of transcripts, how often they were observed, what features were present on each transcript and the location of the dominant transcription start sites and junctions (Additional files 2, 3, 4 and 5: Tables S1- S4). Accurately determining the 5′ end of direct RNAseq transcripts has been shown to be problematic [15, 35, 36]. The accepted model for coronavirus transcription (Fig. 1a) proposes that all viral mRNAs have a common 5′ end [37] and the analysis herein was further restricted to those transcripts that met this criterion. This important step reduces the total number of identified full length subgenomic mRNA molecules to 72,124 and enables an overview of the viral transcriptome of those transcripts that have an authentic 5′ end—transcripts that do not are most likely a result of degradation, a problem with dRNAseq that has also been previously noted [15, 27, 35]. Figure 1c illustrates a transcription map of those transcripts that start at the expected location and have a known ORF as the 5′ most ORF. In Fig. 1c, only the dominant transcript for each ORF is shown; thus, there may be transcripts with structural differences that code for the same ORF but this figure illustrates the structure of the dominant transcript only. This approach was used by us previously to successfully recapitulate the highly complex adenovirus transcriptome de novo [15]. Transcripts that could express all of the predicted ORFs were present, including for the newly predicted ORF10 [3]. However, only one ORF10 transcript was detected, and as previously suggested [9, 10], the status of this as a functional mRNA needs further investigation. Moreover, non-canonical TRS joining events were detected leading to, for example, apparently bone fide subgenomic transcripts coding for ORF7b, as was also recently reported by Kim et al. [10]. Accurate mapping of the junction between the canonical leader and body TRS sequences is problematic as the two sequences are effectively repeated in the viral genome. Mapping algorithms cannot confidently determine a junction breakpoint within repetitive sequences like the TRS sequences, and this is further confounded by the error prone nature of dRNAseq. The full range of identified transcripts (and their structure) that could code for any given ORF is, however, compiled by the pipeline (Additional file 4: Table S3). Moreover, identification and assignment of individual transcripts by our ORF-centric pipeline is not affected by this as it classifies transcripts by 5′ most ORF. Detailed information on the transcripts with a 5′ UTR consistent with the model of coronavirus mRNA expression that would also code for a predicted protein are shown in Table 1. Also reported are the average polyA tail lengths of each transcript group. These are consistent with recently reported findings for SARS-CoV-2 [10].

**Table 1:** Count of transcripts where the 5′ most ORF is a recognised ORF

Feature	Count	Percent of total	Average poly A length of the dominant transcript
Total of all features	72,172		
N	27,882	38.6327	55
None from list	10,453	14.4834	58
M	10,367	14.3642	55
ORF 7a	5162	7.1523	60
ORF 7b	4786	6.6313	61
ORF 3a	3449	4.7788	56
ORF 6	2649	3.6703	56
ORF 8	1447	2.0049	57
E	930	1.2885	56
ORF 9a	530	0.7343	56
S glycoprotein Bristol deletion	86	0.1191	55
S glycoprotein	33	0.0457	54
ORF 3b	6	0.0083	43
ORF 9b	6	0.0083	56
ORF 10	2	0.0027	51

Only transcripts that start to map within the expected leader TRS are considered. For each transcript group, the average polyA length is also shown for the dominant transcript that codes for the indicated ORF. Note that around 14% of transcripts do not apparently code for a known ORF, noted as “none from list”

### Novel deletion in the S glycoprotein

**Fig. 2:**
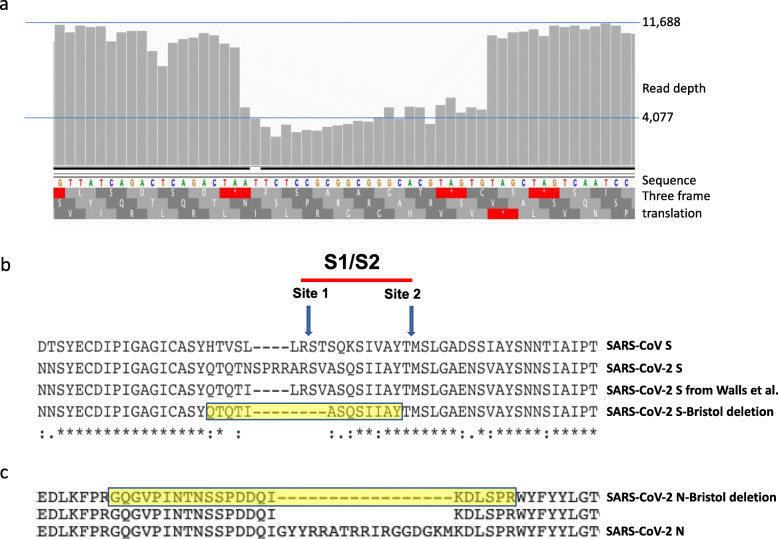
Deletions within the viral mRNAs encoding the S glycoprotein and N protein. **a** The read depth over the region deleted in the S glycoprotein together with information on the sequence in the region and the translation in all three frames. **b** A clustal alignment of four proteins over this region, wild type SARS-CoV, wild type SARS-CoV-2, the artificially deleted version of the wild type SARS-CoV-2 S glycoprotein as reported in Walls et al. [38] and finally the predicted sequence of the deleted protein described here. Highlighted in yellow is the sequence of the unique peptide generated by chymotrypsin digest of the protein which was identified by tandem mass spectrometry. The positions of predicted protease cleavage sites [23] at the S1/S2 boundary are shown. **c** A proposed deletion in the N protein predicted by multiple aligned transcripts and subsequently identified in trypsin digested protein samples as indicated by the unique peptide highlighted in yellow

Manual inspection of the transcripts aligned to the virus genome revealed a large number of reads that aligned to the gene encoding the S glycoprotein but included a 24 nt deletion. This was predicted to result in a deletion of 9 aa, encompassing the proposed furin-like cleavage site (Fig. 2a) with the replacement of a single isoleucine residue. Indeed, there were a large number of transcripts coding for this deleted S glycoprotein that had both a polyA tail > 20 nt and a start at the proposed transcription start location, more than the full length S glycoprotein (Table 1). To confirm this finding and examine if the deletion was present in the original stock samples at the PHE reference laboratory, the region was PCR amplified from the virus passaged at Bristol or from the original stock. These amplified fragments were sequenced using the Sanger method. This revealed that the Bristol stock was indeed a mixture of genomes but the deleted transcript was not detectable in the original stock. Subsequent examination of a very high quality dataset reported recently by Kim et al. [10] did not detect any evidence of similar significant deletions in the S glycoprotein in their data. However, their dataset contained a spontaneous deletion within the region of the genome coding for the E protein resulting in a 9 aa in-frame deletion in almost two thirds of the mapped transcripts (Additional file 6: Figure S1a). Similarly, in one of the datasets reported by Taiaroa et al. [9], there is a 10 nt deletion near the 3′ end of the viral genome outside of any reading frame, the significance of which is unknown (Additional file 6: Figure S1b).

### Proteomic detection of known SARS-CoV-2 proteins

Tandem mass spectrometry was utilised to detect as many viral peptides and phosphopeptides as possible (Additional file 7: Table S5). Table 2 illustrates the numbers of peptides found for each of the predicted ORFs. Notably, whilst most of the ORFs detected were also confirmed in the recent report by Bojkova et al. [11], there were some differences. Whilst we could not detect peptides from ORF9b as described by Bojkova et al., peptides corresponding to the ORF9a protein were identified. This leaves only the E, ORF7b and ORF10 proteins to be detected by mass spectrometry. In addition, peptides unique to proteolytically processed components of the viral replicase polyprotein pp1ab, namely nsp4, nsp9, nsp12, nsp13, nsp14 and nsp15, were identified indicating that these important processing steps occurred as predicted (Table 3).

**Table 2:** Peptide counts for viral proteins

Protein name	Unique peptides	PSMs	Polyprotein 1ab component	Unique peptides	PSMs	Polyprotein 1ab component	Unique peptides	PSMs
**N**	**70**	**4152**	**nsp1**	**10**	**59**	**nsp10**	**5**	**18**
**S glycoprotein**	**76**	**1984**	**nsp2**	**41**	**277**	**nsp12**	**40**	**146**
**Polyprotein 1ab**	**323**	**1816**	**nsp3**	**105**	**850**	**nsp13**	**24**	**70**
**M**	**16**	**250**	**nsp4**	**19**	**105**	**nsp14**	**18**	**34**
**ORF 3a**	**8**	**119**	**nsp5**	**9**	**54**	**nsp15**	**20**	**50**
**ORF 9a**	**14**	**86**	**nsp6**	**5**	**20**	**nsp16**	**13**	**37**
**ORF 8**	**3**	**33**	**nsp7**	**4**	**13**			
**ORF 7a**	**4**	**23**	**nsp8**	**8**	**61**			
**ORF 6**	**2**	**3**	**nsp9**	**4**	**20**			

For each protein, the total number of unique peptides is indicated alongside how many PSMs support the peptides identified. In the case of the viral polyprotein pp1ab, we also list how many peptides uniquely mapped to each nsp region

**Table 3:** Peptides unique to processed proteins

Protein	Contributing PSMs	Sequence identified
**SARS2_pp1ab_nsp4**	**2**	**ALNDFSNSGSDVLYQPPQTSITSAVLQ**
**SARS2_pp1ab_nsp9**	**1**	**NNELSPVALR**
**SARS2_pp1ab__nsp12**	**4**	**SADAQSFLNR**
**SARS2_pp1ab__nsp12**	**1**	**YWEPEFYEAMYTPHTVLQ**
**SARS2_pp1ab__nsp13**	**2**	**AVGACVLCNSQTSLR**
**SARS2_pp1ab__nsp14**	**2**	**AENVTGLFK**
**SARS2_pp1ab__nsp15**	**1**	**SLENVAFNVVNK**

The polyprotein pp1ab is processed into matured smaller proteins nsp1–16 during infection; this table indicates which unique peptides were identified that could only arise as a result of full polyprotein processing

Initially, no unique peptides corresponding to the novel deleted version of the S glycoprotein were detected by tandem mass spectrometry. However, a typical tryptic digest of this region would generate a very large peptide which would not be detectable within the standard mass range used for analysis. Accordingly, samples were digested using chymotrypsin and the analysis repeated revealing a single spectra which matched to the peptide corresponding to the deleted version of the S-glycoprotein, but only when using a 5% false discovery threshold. However, this did provide an accurate *m/z* ratio and ionisation status for this peptide, enabling a targeted search which identified nine PSM’s with high confidence (i.e. FDR 1%) that corroborate the expression of the deleted spike protein (Additional file 6: Figure S2 and Additional file 8: Table S6).

### Proteomic detection of previously unknown viral proteins

In Table 1, we note that approximately 14% of transcripts do not code for a known ORF and are collated under the heading “none from list”. In principle, this is a large body of transcripts that could code for novel proteins. As we have shown before, transcriptomics and proteomics can be combined to explore this question more deeply and provide an unbiased evaluation of the proteome of viruses and higher eukaryotes [12, 13, 39]. We used our ORF-centric pipeline to remove errors in the nanopore sequence data and generate a list of possible ORFs by translating the first ORF present on every transcript sequenced [15].

Using this list of predicted, and potentially novel, proteins revealed a large number of possible proteins which do not correspond to the standard list of predicted viral proteins. In the majority of cases, they appeared to be transcripts with rare and unusual structures likely resulting from rare rearrangements of the viral genome or potentially during subgenomic mRNA synthesis. However, in some cases, the translation of specific classes of relatively abundant transcripts was supported by direct peptide evidence. In particular, multiple versions of the N protein with distinct small internal deletions were detected and more than 20 transcripts that could encode for these proteins were also identified (Additional file 9: Table S7). A schematic of such a deletion in the N protein is shown, indicating the peptide that was identified by tandem mass spectrometry to support the observed transcripts (Fig. 2c).

### Phosphoproteomic analysis of SARS-CoV-2 proteins

**Fig. 3:**
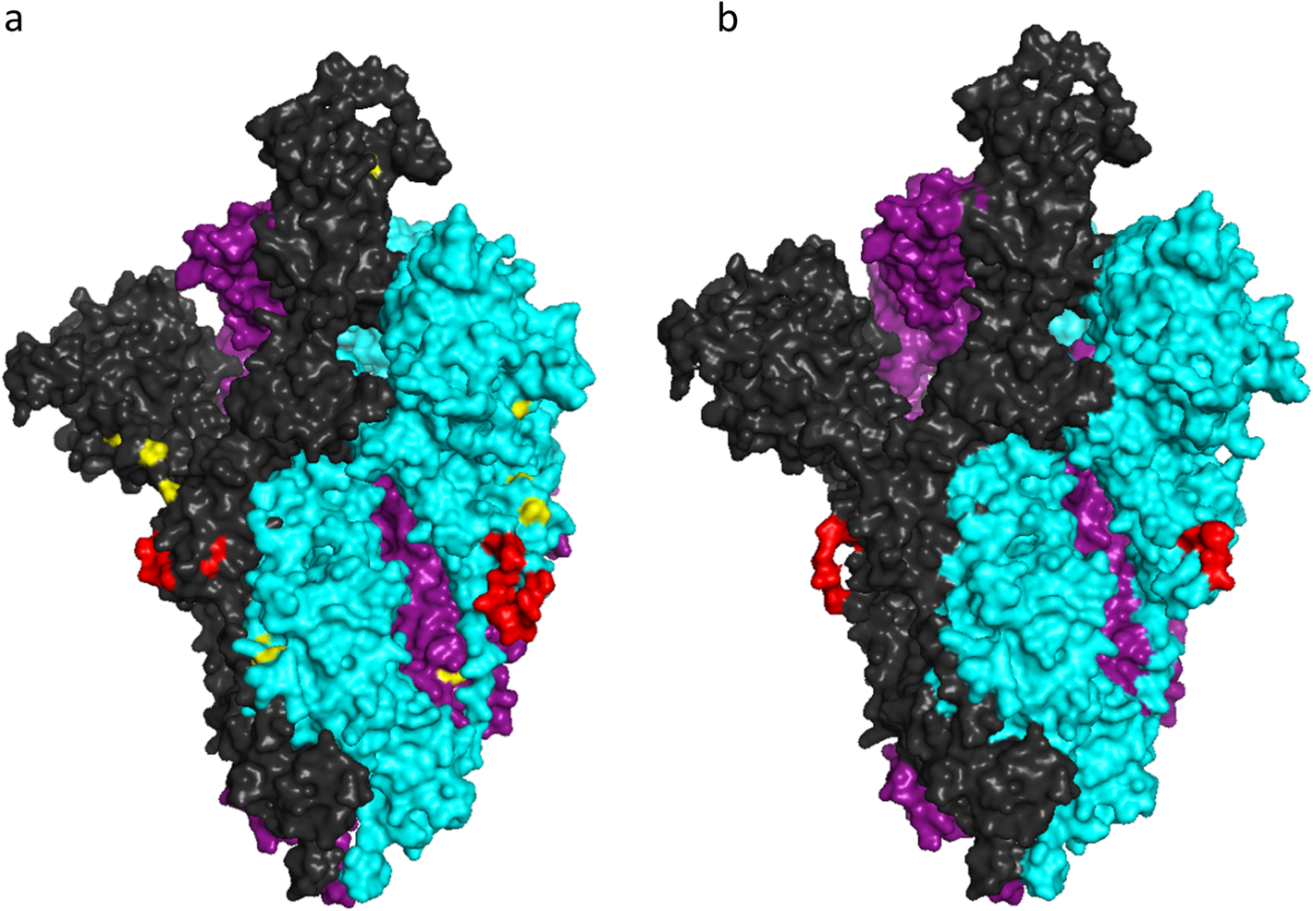
A space filled model of the wild type SARS-CoV-2 S glycoprotein in a trimeric form using the sequence of **a** the native or **b** spike deletant virus, in which the aa’s _679_NSPRRARSV_687_ have been replaced with isoleucine. The model was built using a cryo-EM structure (6VSB.pdb) of the S glycoprotein in the prefusion form (25). Each of the monomers is coloured differently. The loop containing the furin cleavage site (or the shortened loop in the deleted version in **b**) is indicated in red. The positions of phosphorylation sites identified by mass spectrometry and surface located were mapped on the native structure and shown in yellow in **a**

**Fig. 4:**
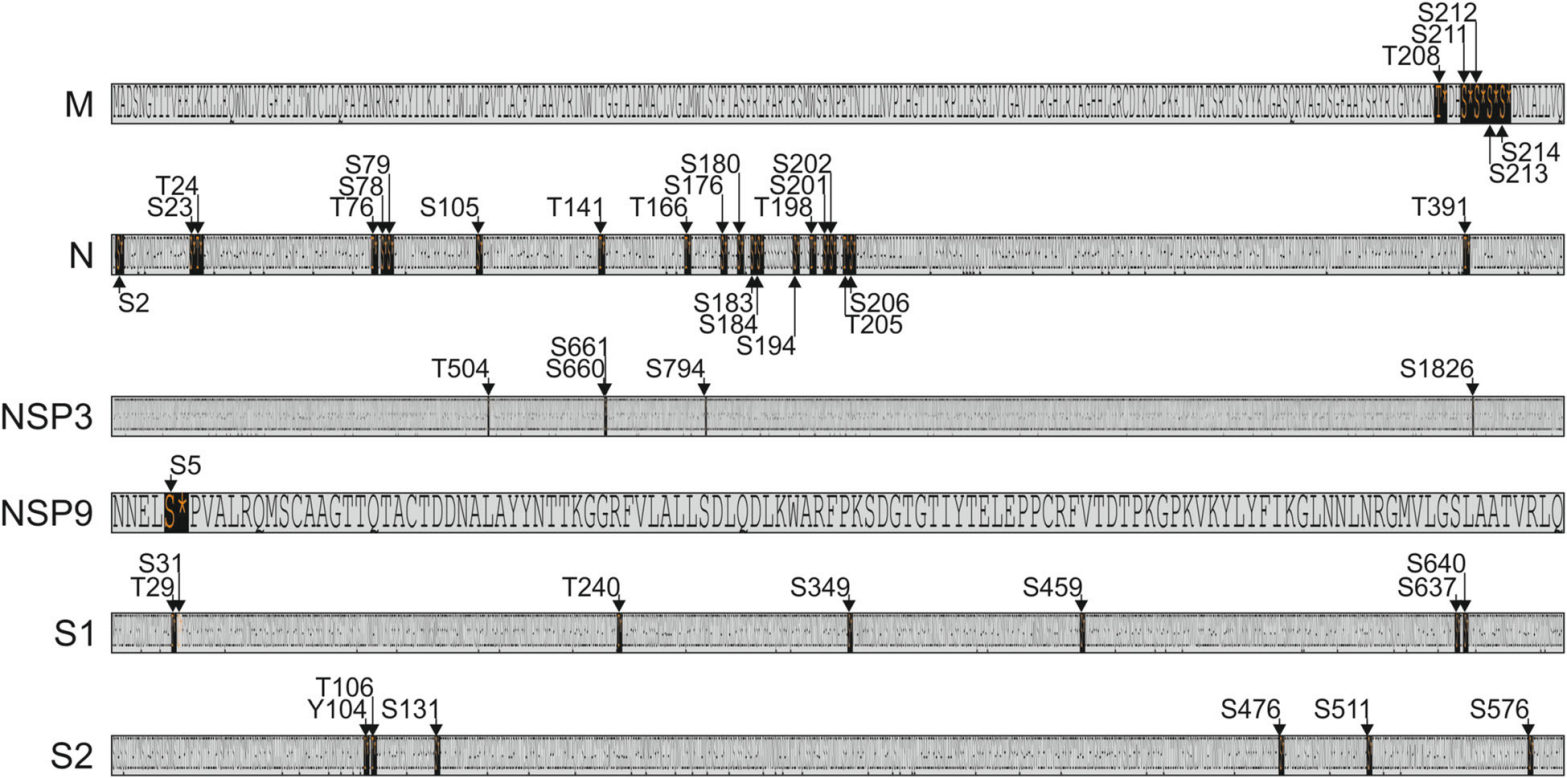
Schematic of the location of phosphorylation sites. Proteins M, N, NSP3, NSP9 and S glycoprotein are shown as we have accurate phospho-site data for these proteins. For each location, we indicate the amino acids (S, T or Y) and the amino acid numbering. The S glycoprotein is shown as S1 and S2 to illustrate where the sites would be relative to the major cleavage site on the wild type S glycoprotein

**Fig. 5:**
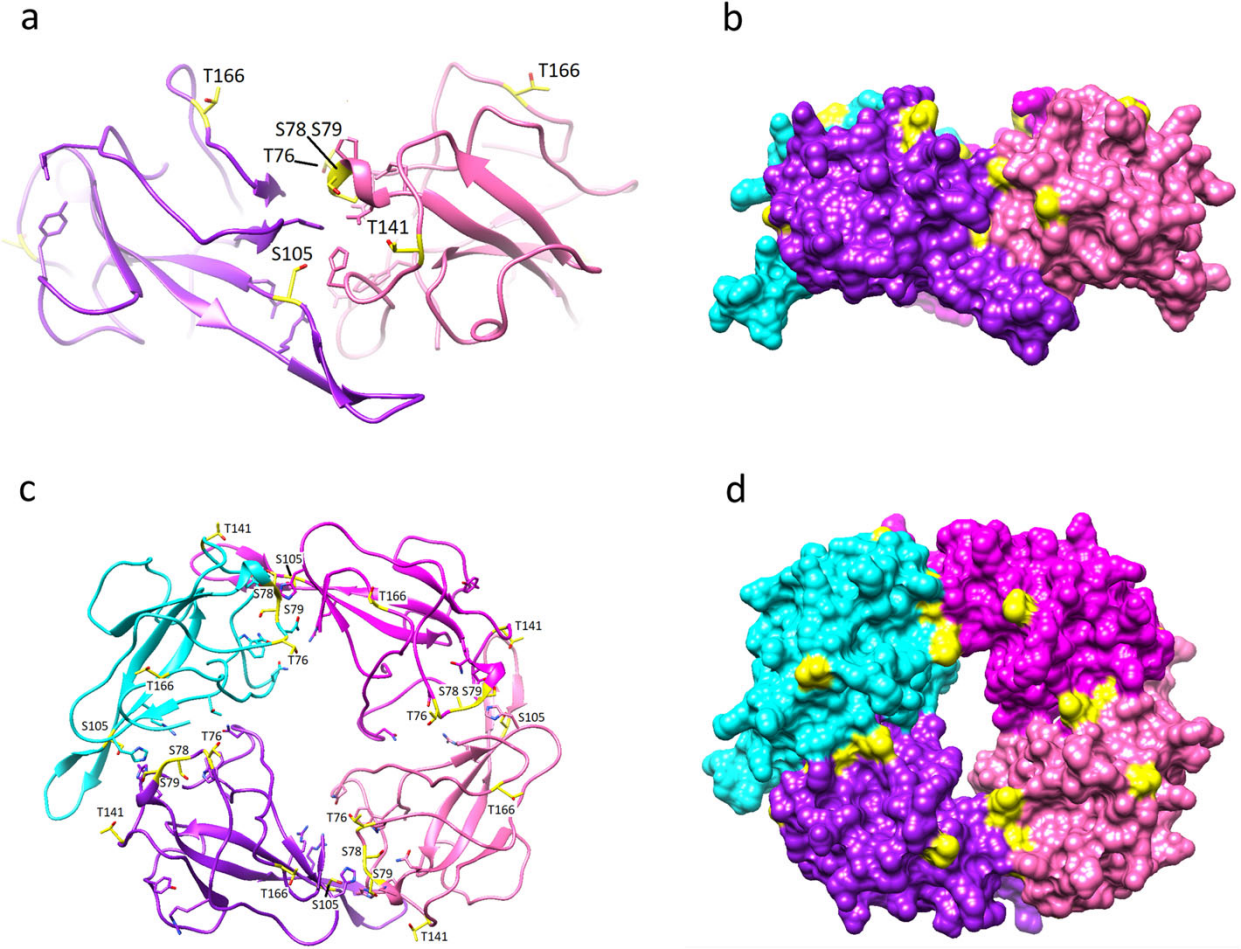
Modelling phosphorylation on the RNA binding domain of N protein. The positions of phosphorylation sites identified by mass spectrometry were mapped on the x-ray crystal structure of the N-terminal RNA binding domain of the N protein (aa residues 47–173) from SARS-CoV-2 (6YVO.pdb). The four monomer units in one asymmetric unit are distinctly coloured and shown as side (**a**, **b**) and top (**c**, **d**) views as ribbon (left hand figures) and space filling models (right hand figures)

Allied to an analysis of viral peptides, the phosphorylation status of viral proteins was investigated as this could reveal potential targets for licenced kinase inhibitors. Phosphopeptides corresponding to locations on the N, M, ORF 3a, nsp3, nsp9, nsp12 and S glycoprotein were detected (Table 4). The detection of phosphorylation sites on the S glycoprotein has not been previously noted and may be of significance in the context of vaccines based on this protein; the location of these sites is illustrated (Fig. 3). The location of the phosphorylation sites on the proteins listed is illustrated in Fig. 4, and additional models of the phosphorylation sites on the N-terminal RNA-binding domain of the N protein are shown in Fig. 5.

**Table 4:** Phosphopeptide counts

Protein	Number of distinct identified phosphorylation sites	Location of phospho sites	Number of contributing PSMs
N	20	S2, S105, T141, S176, S180, S183, S184, T391, S78, S79, T76, S206, T205, S23, T24, T166, S194, S201, S202, T198	74
M	5	S211, S212, T208, S213, S214	43
S glycoprotein	13	S1261, S1161, S1196, T791, Y789, S459, S816, S349, T240, S31, T29, S637, S640	21
nsp3	5	S794, S661, T504, S1826, S660	16
ORF 3a	0	QGEIKDATPSDFVR	1
nsp9	1	S5	1
nsp12	0	GFFKEGSSVELK	1

Listed for each protein is the number of distinct phospho sites identified along with the locations and amino acid modified as well as how many contributing PSMs there are in total. Each named site has confidence of at least 70% as defined by the PhosphoRS node of Proteome Discoverer software. For proteins ORF3a and nsp12, no distinct site could be identified despite a phosphorylated peptide being found; in these cases, the peptide sequence is provided

## Discussion

This integrated analysis of the SARS-CoV-2 transcriptome and proteome revealed significant findings, in particular, the identification of an eight aa deletion in the SARS-CoV-2 S glycoprotein that potentially effects protein cleavage, cell tropism and infectivity. The coronavirus S glycoprotein is made as a larger precursor that must be primed by cleavage using host cell proteases to enable subsequent viral entry [40]. For different coronaviruses, cleavage can occur at one or more sites (termed the S1/S2 and S2′ sites) depending on the host cell and the amino acid consensus sequences present at each site, with major repercussions on host cell tropism and pathogenesis [20]. The SARS-CoV S glycoprotein has a single arginine residue at the S1/S2 site (Fig. 2b) that facilitates cleavage by a number of proteases including trypsin, cathepsin L and TMPRSS11D [41–43] and a consensus sequence for trypsin and/or elastin cleavage at the S2′ site [21, 44]. By comparison, SARS-CoV-2 has a four amino acid insertion (_680_SPRR_683_) in the S glycoprotein that results in the generation of a furin-like cleavage site (_682_RRAR_685_) at the S1/S2 boundary that is not present in other lineage B betacoronaviruses, including SARS-CoV and the highly related bat coronavirus RaTG13 [23]. Moreover, analysis of a cryo-EM structure of the S glycoprotein showed that the SPRR insertion creates a disordered solvent exposed loop [24] that protrudes from the trimer surface (Fig. 3a). It has been proposed that this region is more available to host cell proteases for processing [24]. The presence of a furin-like cleavage site at the S1/S2 boundary results in cleavage of the SARS-CoV-2 S glycoprotein before viral exit from the cell, whereas the S glycoprotein of coronaviruses such as SARS-CoV, lacking a S1/S2 furin cleavage site, exit the cells with the S glycoprotein largely uncleaved, necessitating cleavage prior to or during cell entry [21, 38, 45].

The SARS-CoV-2 S glycoprotein deletion identified in this study removes the furin cleavage site, but also Arg_685_ (corresponding to SARS-CoV Arg_667_) required for cleavage of the SARS-CoV S glycoprotein. However, the sequence _694_AYT/M_697_ (numbering of undeleted S glycoprotein, cleavage shown by “/”; Fig. 2b) is still retained and has been identified as a second potential S1/S2 protease cleavage site for SARS-CoV downstream of the basic Arg_667_ residue [23, 42] suggesting that either the deleted SARS-CoV-2 S glycoprotein could be cleaved at this site or only use the S2′ site for cleavage. Recently, pseudoviruses expressing the SARS-CoV-2 S glycoprotein and the corresponding S glycoprotein with the furin cleavage site removed at the S1/S2 boundary (residues _680_SPRR_683_ removed) were made and their ability to mediate entry to Vero E6 and BHK-21 cells expressing hACE2 compared [38]. Deletion of the furin cleavage site enhanced the entry of the corresponding pseudovirus into Vero E6 cells but diminished entry into hACE2 BHK-21 cells. Although the engineered deletion differed from the naturally occurring deletion described here, this observation suggests that the naturally occurring deletion (in cell culture) enhances the ability of the virus to enter Vero cells and was selected for during passage in Vero E6 cells, a cell line that lacks a working type I interferon response. This has clear implications for the use of Vero cells to propagate and grow large batches of the virus for research and especially virus batches grown for use in vaccine challenge studies. Moreover, it also raises the possibility that even virus stocks which have been carefully assayed for homogeneity could still spontaneously generate this deletion during animal challenge studies—particularly in non-human primates and perhaps especially in animals from the *Chlorocebus* genus. Thus, vaccine studies will need to carefully monitor the homogeneity of the challenge virus throughout the study period. Indeed, the functional impact of this kind of deletion is already beginning to emerge from recent preprint data. For example, viruses carrying the deletion in the S glycoprotein reported in this study may well be attenuated in vivo in NHPs as this type of furin deletion virus has been shown to exhibit reduced pathogenicity in a hamster disease model [46]. The early emergence of a viral population carrying a deletion in the S glycoprotein that removes the furin cleavage site within an individual challenge animal could give the false impression that an individual animal had a mild infection. Given that animal studies are required to use the smallest number of animals needed for statistical significance, just one such event could confound the study. Since our initial findings were reported by pre-print archive, other teams have detected deletions in the S glycoprotein that remove the furin cleavage site at low levels alongside wild type S glycoprotein sequences in clinical samples [47]. Other teams have also reported the emergence of this type of deletion and other mutations at this location in independent cell culture based experiments [48]. This independent cell culture passage analysis concluded that viruses containing deletions in the S glycoprotein, that removed the furin cleavage site, result in a large plaque phenotype in Vero E6 cells and that this mutation is strongly and frequently selected for in Vero E6 cells. These observations raise the potential that in vivo, there are a subset of host cells where the deletion variant has a selectable advantage over the wild type. However, it is important to note that even where these types of deletions have been reported in clinical samples, they do not appear to dominate in vivo as there is still relatively little global variation reported in viral genomes including the S glycoprotein at this time [49].

In addition to the S glycoprotein deletion, we were also able to find an additional significant deletion, in high-quality data recently deposited by Kim et al. [10], in the region of the E protein. This deletion removes one of the primer sites recently recommended as a first-line screening tool [50]. This finding reinforces the potential of the virus to incur unexpected spontaneous deletions in viral genes during passaging that are hard to predict and detect. A recent analysis showed that deletion of ORF8 from human clinical isolates is possible [51] and our data shows that novel SARS-CoV-2 viruses containing deletions or even insertions can arise naturally and successfully propagate. As the virus continues to spread and potentially comes under selective pressure from the host response in humans, vaccines and antiviral drugs in the future, vigilance will be required to detect novel rearrangements/deletions. Coronaviruses use a distinctive complex mechanism for genome replication that needs to be considered during bioinformatic analysis of their genomes relative to viruses causing other epidemics and pandemics where the principle driver of viral change would be single nucleotide polymorphisms and/or small (< 3 nt) insertions/deletions. If the plasticity of the SARS-CoV-2 genome follows the same pathway as HCoV-NL63 [52], then different isolates will emerge through recombination and this may result in repeat infection as protection will not be effective.

The detection of numerous phosphorylation sites within critical viral proteins is another key resource. The phosphorylation of the N protein of multiple coronaviruses is well known [53–57], and the phosphorylation sites identified lie in either the N-terminal RNA-binding domain or interdomain linker [58]. Mapping of the phosphorylation sites on an x-ray structure of the SARS-CoV-2 N-terminal RNA-binding domain showed they were surface located (Fig. 5). However, we believe this is the first report providing evidence of phosphorylation of the coronavirus proteins M, nsp3 and S. These are all membrane bound proteins. M protein is critical in forming the viral particle, nsp3 is a key multifunctional component of the replication/transcription machinery and S protein is the major attachment protein respectively [59]. The phosphorylation sites identified on the S glycoprotein may be important in assembly of the trimer. Residues T29, S31, S349, T791 and S816 are all surface located, whilst T240 sits underneath a disordered loop which when phosphorylated will add negative charge which may influence loop conformation. Residues Y789 and (perhaps to a lesser extent) T791 sit at the subunit interface and may be involved in controlling trimer assembly. S637 and S640 lie in a modelled loop but are nevertheless potentially interesting. They are close together in sequence and in the loops modelled for the compact folds these model well as hairpins. In the chain with the extended domain, the same loop models in an extended form. This would be consistent with phosphorylation at S637 and S640 forcing the hairpin apart. Thus, these amino acids are worth exploring as potential control points for spike conformational changes from compact to an extended form. Overall, the identification of protein phosphorylation sites is notable but some caveats are needed here and of course the status and functional significance of these sites will take considerable time to independently validate and investigate by point mutation and/or biochemical techniques. However, identifying phosphorylation sites on multiple viral proteins is a valuable starting point for rational investigation of clinically licenced kinase inhibitors as antiviral drugs as has previously been observed for SARS-CoV [60].

Transcriptomically, our findings broadly agree with those recently reported [9, 10]. Our assessment of polyA tail length is in line with both the data reported for SARS-CoV-2 [10] and for data on bovine coronavirus [61] although the role and significance of this data is unclear in either viral system. One area of difference is in the abundance of each transcript where we disagree with Kim et al. [10]; however, our ORF-centric pipeline assessment of individual transcript abundance does broadly agree with Taiaroa et al. [9] and the recent report of SARS-CoV-2 mRNA abundance determined by Northern blot [48].

## Conclusions

This integrated transcriptomic and proteomic dataset is a rich resource for research teams building a picture of this novel virus as it enables an accurate overview of the transcription profile and adds significant new direct observational data for the viral proteins. Critically, it provides direct observation of both transcripts and proteins and potential phosphorylation sites and highlights this virus’s potential to mutate via genomic recombination/deletions. This latter observation has potential to directly affect vaccine challenge studies and may have a role to play as the pandemic progresses and vaccines or antivirals are widely deployed.

## Supplementary information

**Additional file 1.** A text file delimitating the locations of known features on the SARS-CoV-2 genome.

**Additional file 2: Table S1.** Transcripts coding for an ORF count table. A table of the number of times transcripts were detected that would code for any given ORF. This considers only transcripts that start within the leader region of the viral genome.

**Additional file 3: Table S2.** Transcript structure and ORF assignment table. A table outlining the distinct structures of transcripts, how many times a particular type of transcript was detected and which ORFS the transcripts could code for. Only transcripts that start within the leader TRS are considered.

**Additional file 4: Table S3.** Transcript structure and ORF assignment table. Similar to supplementary 2, a table outlining the distinct structures of transcripts, how many times a particular type of transcript was detected and which ORFS the transcripts could code for. In this case all transcripts are listed irrespective of where the transcription start is mapped to.

**Additional file 5: Table S4.** Start locations and internal transcript boundaries. A list of the number of ties a genome location is used as a transcript start, transcript stop or is noted at the location of a transcript boundary (akin to an exon-intron boundary).

**Additional file 6: Figures S1 and S2.** Supplementary figures detailing in Figure S1 the location of deletions in other reported direct RNAseq data and in Figure S2 the MS/MS spectra for the peptide unique to the furing cleavage site deletion variant.

**Additional file 7: Table S5.** A list of unique peptides detected by MS/MS and their parent protein.

**Additional file 8: Table S6.** Peptide identification report from MS/MS based search of Chymotrypsin digested proteins.

**Additional file 9: Table S7.** Location and alignments of N protein peptides resulting from rare deletions within N gene.

## Data Availability

The fastq files and ThermoFisher .raw files are available on zenodo.org under the following dois: 10.5281/zenodo.3722580 for the fastq data [28], 10.5281/zenodo.3722604 for the phosphoproteomics .RAW files [29], 10.5281/zenodo.3722590 for the total proteome .RAW files (slices 1–10 of 20) [30] and 10.5281/zenodo.3722596 for the total proteome .RAW files (slices 11–20 of 20) [31]. The proteomics data has also been deposited at PRIDE (PXD018241) [32] and the transcriptomics data at the European Nucleotide Archive (PRJEB39337) [33].

## References

[1] Dong E, Du H, Gardner L. An interactive web-based dashboard to track COVID-19 in real time. Lancet Infect Dis. 2020. 10.1016/S1473-3099(20)30120-1. 10.1016/S1473-3099(20)30120-1 PMC7159018 32087114

[2] Kottier SA, Cavanagh D, Britton P (1995). Experimental evidence of recombination in coronavirus infectious bronchitis virus. Virology.

[3] Wu F (2020). A new coronavirus associated with human respiratory disease in China. Nature.

[4] Zhou P (2020). A pneumonia outbreak associated with a new coronavirus of probable bat origin. Nature.

[5] Perlman S, Netland J (2009). Coronaviruses post-SARS: update on replication and pathogenesis. Nat Rev Microbiol.

[6] Irigoyen N (2016). High-resolution analysis of coronavirus gene expression by RNA sequencing and ribosome profiling. PLoS Pathog.

[7] Hiscox JA, Mawditt KL, Cavanagh D, Britton P (1995). Investigation of the control of coronavirus subgenomic mRNA transcription by using T7-generated negative-sense RNA transcripts. J Virol.

[8] Snijder EJ (2003). Unique and conserved features of genome and proteome of SARS-coronavirus, an early split-off from the coronavirus group 2 lineage. J Mol Biol.

[9] Taiaroa G, et al. Direct RNA sequencing and early evolution of SARS-CoV-2. bioRxiv, 2020.2003.2005.976167. 2020. 10.1101/2020.03.05.976167.

[10] Kim D, et al. The architecture of SARS-CoV-2 transcriptome. Cell. 2020. 10.1016/j.cell.2020.04.011. 10.1016/j.cell.2020.04.011 PMC7179501 32330414

[11] Denisa Bojkova KK, Koch B, Widera M, Krause D, Ciesek S, Cinatl J, Münch C. SARS-CoV-2 infected host cell proteomics reveal potential therapy targets. Nature. 2020. 10.21203/rs.3.rs-17218/v1. 10.1038/s41586-020-2332-7 PMC7616921 32408336

[12] Evans VC (2012). De novo derivation of proteomes from transcriptomes for transcript and protein identification. Nat Methods.

[13] Wynne JW (2014). Proteomics informed by transcriptomics reveals Hendra virus sensitizes bat cells to TRAIL-mediated apoptosis. Genome Biol.

[14] Aljabr W, et al. High resolution analysis of respiratory syncytial virus infection in vivo. Viruses. 2019;11. 10.3390/v11100926. 10.3390/v11100926 PMC6832471 31658630

[15] Donovan-Banfield I, Turnell AS, Hiscox JA, Leppard KN, Matthews DA (2020). Deep splicing plasticity of the human adenovirus type 5 transcriptome drives virus evolution. Commun Biol.

[16] Li F (2016). Structure, function, and evolution of coronavirus spike proteins. Annu Rev Virol.

[17] Tortorici MA, Veesler D (2019). Structural insights into coronavirus entry. Adv Virus Res.

[18] Bosch BJ, van der Zee R, de Haan CAM, Rottier PJM (2003). The coronavirus spike protein is a class I virus fusion protein: structural and functional characterization of the fusion core complex. J Virol.

[19] Kirchdoerfer RN (2016). Pre-fusion structure of a human coronavirus spike protein. Nature.

[20] Millet JK, Whittaker GR (2015). Host cell proteases: critical determinants of coronavirus tropism and pathogenesis. Virus Res.

[21] Belouzard S, Chu VC, Whittaker GR (2009). Activation of the SARS coronavirus spike protein via sequential proteolytic cleavage at two distinct sites. Proc Natl Acad Sci U S A.

[22] Madu IG, Roth SL, Belouzard S, Whittaker GR (2009). Characterization of a highly conserved domain within the severe acute respiratory syndrome coronavirus spike protein S2 domain with characteristics of a viral fusion peptide. J Virol.

[23] Coutard B (2020). The spike glycoprotein of the new coronavirus 2019-nCoV contains a furin-like cleavage site absent in CoV of the same clade. Antivir Res.

[24] Wrapp D (2020). Cryo-EM structure of the 2019-nCoV spike in the prefusion conformation. Science.

[25] Andersen KG, Rambaut A, Lipkin WI, Holmes EC, Garry RF. The proximal origin of SARS-CoV-2. Nat Med. 2020. 10.1038/s41591-020-0820-9. 10.1038/s41591-020-0820-9 PMC7095063 32284615

[26] REED LJ, MUENCH H (1938). A simple method of estimating fifty per cent endpoints12. Am J Epidemiol.

[27] Workman RE (2019). Nanopore native RNA sequencing of a human poly(A) transcriptome. Nat Methods.

[28] Davidson AD, et al. Characterisation of the transcriptome and proteome of SARS-CoV-2 reveals a cell passage induced in-frame deletion of the furin-like cleavage site from the spike glycoprotein. FASTQ dataset. Zenodo. 2020. 10.5281/zenodo.3722580. 10.1186/s13073-020-00763-0 PMC7386171 32723359

[29] Davidson AD, et al. Characterisation of the transcriptome and proteome of SARS-CoV-2 reveals a cell passage induced in-frame deletion of the furin-like cleavage site from the spike glycoprotein. Phosphoprotoemics dataset. Zenodo. 2020. 10.5281/zenodo.3722604. 10.1186/s13073-020-00763-0 PMC7386171 32723359

[30] Davidson AD, et al. Characterisation of the transcriptome and proteome of SARS-CoV-2 reveals a cell passage induced in-frame deletion of the furin-like cleavage site from the spike glycoprotein. proteomics (1-10 of 20) dataset. Zenodo. 2020. 10.5281/zenodo.3722590. 10.1186/s13073-020-00763-0 PMC7386171 32723359

[31] Davidson AD, et al. Characterisation of the transcriptome and proteome of SARS-CoV-2 reveals a cell passage induced in-frame deletion of the furin-like cleavage site from the spike glycoprotein. Proteomics dataset (11-20 of 20). Zenodo. 2020. 10.5281/zenodo.3722596. 10.1186/s13073-020-00763-0 PMC7386171 32723359

[32] Davidson, A. D. *et al.* Characterisation of the transcriptome and proteome of SARS-CoV-2 reveals a cell passage induced in-frame deletion of the furin-like cleavage site from the spike glycoprotein. Proteomics dataset. PRIDE PXD018241. 2020. 10.1186/s13073-020-00763-0 PMC7386171 32723359

[33] Davidson, A. D. *et al.* Characterisation of the transcriptome and proteome of SARS-CoV-2 reveals a cell passage induced in-frame deletion of the furin-like cleavage site from the spike glycoprotein. Transcriptomics data. ENA PRJEB39337. 2020. 10.1186/s13073-020-00763-0 PMC7386171 32723359

[34] Viehweger A (2019). Direct RNA nanopore sequencing of full-length coronavirus genomes provides novel insights into structural variants and enables modification analysis. Genome Res.

[35] Depledge DP (2019). Direct RNA sequencing on nanopore arrays redefines the transcriptional complexity of a viral pathogen. Nat Commun.

[36] Garalde DR (2018). Highly parallel direct RNA sequencing on an array of nanopores. Nat Methods.

[37] Sawicki SG, Sawicki DL, Siddell SG (2007). A contemporary view of coronavirus transcription. J Virol.

[38] Walls AC, et al. Structure, function, and antigenicity of the SARS-CoV-2 spike glycoprotein. Cell. 2020. 10.1016/j.cell.2020.02.058. 10.1016/j.cell.2020.02.058 PMC7102599 32155444

[39] Maringer K (2017). Proteomics informed by transcriptomics for characterising active transposable elements and genome annotation in Aedes aegypti. BMC Genomics.

[40] Letko M, Marzi A, Munster V. Functional assessment of cell entry and receptor usage for SARS-CoV-2 and other lineage B betacoronaviruses. Nat Microbiol. 2020. 10.1038/s41564-020-0688-y. 10.1038/s41564-020-0688-y PMC7095430 32094589

[41] Li F (2006). Conformational states of the severe acute respiratory syndrome coronavirus spike protein ectodomain. J Virol.

[42] Bosch BJ, Bartelink W, Rottier PJM (2008). Cathepsin L functionally cleaves the severe acute respiratory syndrome coronavirus class I fusion protein upstream of rather than adjacent to the fusion peptide. J Virol.

[43] Bertram S (2011). Cleavage and activation of the severe acute respiratory syndrome coronavirus spike protein by human airway trypsin-like protease. J Virol.

[44] Belouzard S, Madu I, Whittaker GR (2010). Elastase-mediated activation of the severe acute respiratory syndrome coronavirus spike protein at discrete sites within the S2 domain. J Biol Chem.

[45] Hoffmann M, et al. SARS-CoV-2 cell entry depends on ACE2 and TMPRSS2 and is blocked by a clinically proven protease inhibitor. Cell. 2020. 10.1016/j.cell.2020.02.052. 10.1016/j.cell.2020.02.052 PMC7102627 32142651

[46] Lau S-Y, et al. Attenuated SARS-CoV-2 variants with deletions at the S1/S2 junction. Emerg Microbes Infect. 2020:1–15. 10.1080/22221751.2020.1756700. 10.1080/22221751.2020.1756700 PMC7241555 32301390

[47] Liu Z, et al. Identification of a common deletion in the spike protein of SARS-CoV-2. bioRxiv, 2020.2003.2031.015941. 2020. 10.1101/2020.03.31.015941.

[48] Ogando NS, et al. SARS-coronavirus-2 replication in Vero E6 cells: replication kinetics, rapid adaptation and cytopathology. J Gen Virol, 2020.2004.2020.049924. 2020. 10.1099/jgv.0.001453. 10.1099/jgv.0.001453 PMC7654748 32568027

[49] MacLean OA, Orton RJ, Singer JB, Robertson DL. No evidence for distinct types in the evolution of SARS-CoV-2. Virus Evol. 2020;6. 10.1093/ve/veaa034. 10.1093/ve/veaa034 PMC7197565 32817804

[50] Corman VM, et al. Detection of 2019 novel coronavirus (2019-nCoV) by real-time RT-PCR. Euro Surveill. 2020;25. 10.2807/1560-7917.ES.2020.25.3.2000045. 10.2807/1560-7917.ES.2020.25.3.2000045 PMC6988269 31992387

[51] Su YC, et al. Discovery of a 382-nt deletion during the early evolution of SARS-CoV-2. bioRxiv, 2020.2003.2011.987222. 2020. 10.1101/2020.03.11.987222.

[52] Kiyuka PK (2018). Human coronavirus NL63 molecular epidemiology and evolutionary patterns in rural coastal Kenya. J Infect Dis.

[53] McBride R, van Zyl M, Fielding BC (2014). The coronavirus nucleocapsid is a multifunctional protein. Viruses.

[54] Wu CH, Chen PJ, Yeh SH (2014). Nucleocapsid phosphorylation and RNA helicase DDX1 recruitment enables coronavirus transition from discontinuous to continuous transcription. Cell Host Microbe.

[55] Peng TY, Lee KR, Tarn WY (2008). Phosphorylation of the arginine/serine dipeptide-rich motif of the severe acute respiratory syndrome coronavirus nucleocapsid protein modulates its multimerization, translation inhibitory activity and cellular localization. FEBS J.

[56] Chen H (2005). Mass spectroscopic characterization of the coronavirus infectious bronchitis virus nucleoprotein and elucidation of the role of phosphorylation in RNA binding by using surface plasmon resonance. J Virol.

[57] Spencer KA, Dee M, Britton P, Hiscox JA (2008). Role of phosphorylation clusters in the biology of the coronavirus infectious bronchitis virus nucleocapsid protein. Virology.

[58] Kang S, et al. Crystal structure of SARS-CoV-2 nucleocapsid protein RNA binding domain reveals potential unique drug targeting sites. bioRxiv, 2020.2003.2006.977876. 2020. 10.1101/2020.03.06.977876. 10.1016/j.apsb.2020.04.009 PMC7194921 32363136

[59] Fehr AR, Perlman S (2015). Coronaviruses: an overview of their replication and pathogenesis. Methods Mol Biol.

[60] Wu CH (2009). Glycogen synthase kinase-3 regulates the phosphorylation of severe acute respiratory syndrome coronavirus nucleocapsid protein and viral replication. J Biol Chem.

[61] Wu H-Y, Ke T-Y, Liao W-Y, Chang N-Y (2013). Regulation of coronaviral poly(A) tail length during infection. PLoS One.

