# Supplementary material for: Characterisation of the transcriptome and proteome of SARS-CoV-2 reveals a cell passage induced in-frame deletion of the furin-like cleavage site from the spike glycoprotein
Source: Genome Med. 2020 Jul 28;12:68. doi: 10.1186/s13073-020-00763-0 (PMC7386171; doi:10.1186/s13073-020-00763-0)
Supplement: Supplementary file 6 — Additional file 6: Figures S1 and S2. Supplementary figures detailing in Figure S1 the location of deletions in other reported direct RNAseq data and in Figure S2 the MS/MS spectra for the peptide unique to the furing cleavage site deletion variant. [file 13073_2020_763_MOESM6_ESM.pdf]

Supplementary Figure 1

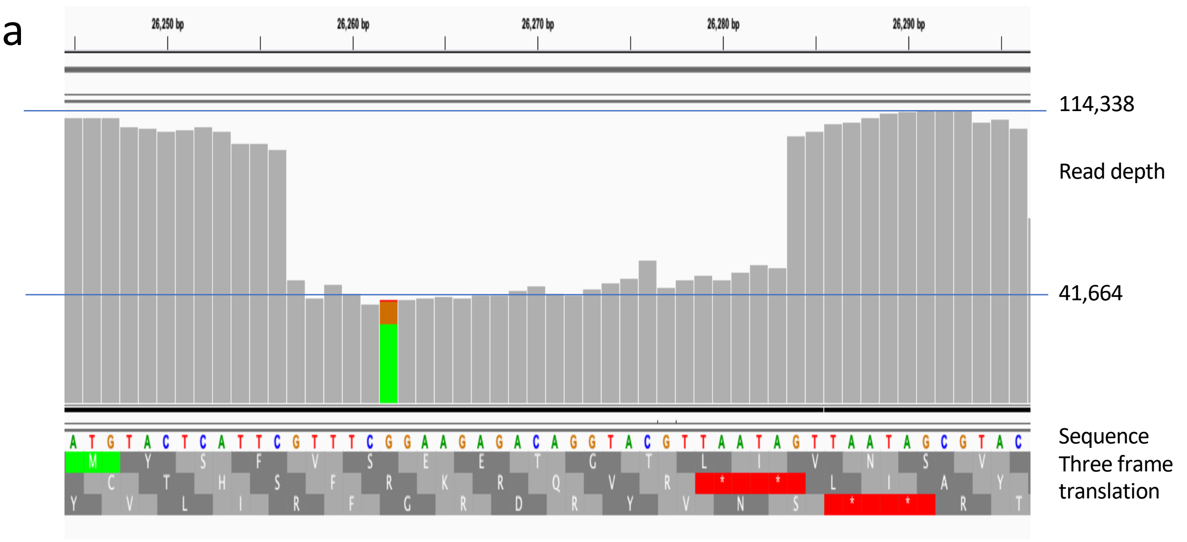

IGV viewer illustration of 27 nt deletion in the viral transcripts covering the beginning of the region coding for envelope E protein from Kim et al.[9] The initiating methionine is seen in the top row of the three frame translation.

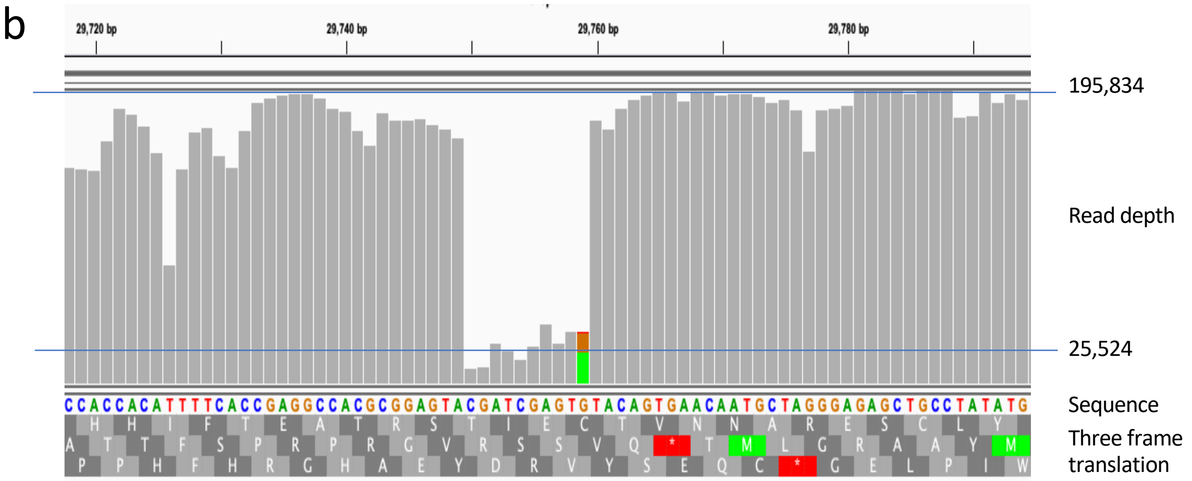

IGV viewer illustration of 10 nt deletion in the viral transcripts from the Taiaros et al.[10] dataset named “covid_update.fastq” mapped to the wuhan-Hu-1 genome. There are no ORFs affected by this deletion as there are stop codons in all three frames upstream of this deletion.

Supplementary Figure 2

Peptide Summary

Sequence: QTQTIASQSIIAY, Charge: +2, Monoisotopic m/z: 712.37494 Da (-0.07 mmu/-0.09 ppm), MH+: 1423.74260 Da, RT: 90.5494 min,
Identified with: Sequest HT (v1.17); XCorr:3.75, Ions matched by search engine: 0/0
Fragment match tolerance used for search: 0.6 Da
Fragments used for search: b; b-H_2O; b-NH_3; y; y-H_2O; y-NH_3

Fragment Matches

Value Type: Theo. Mass [Da]

Ion Series | Neutral Losses | Precursor Ions

#1	b^+	b^{2+}	Seq.	y^+	y^{2+}	#2
1	129.06585	65.03657	Q			13
2	230.11353	115.56040	T	1295.68416	648.34572	12
3	358.17211	179.58969	Q	1194.63648	597.82188	11
4	459.21979	230.11353	T	1066.57790	533.79259	10
5	572.30385	286.65556	I	965.53022	483.26875	9
6	643.34097	322.17412	A	852.44616	426.72672	8
7	730.37299	365.69014	S	781.40905	391.20816	7
8	858.43157	429.71942	Q	694.37702	347.69215	6
9	945.46360	473.23544	S	566.31844	283.66286	5
10	1058.54766	529.77747	I	479.28641	240.14684	4
11	1171.63173	586.31950	I	366.20235	183.60481	3
12	1242.66884	621.83806	A	253.11828	127.06278	2
13			Y	182.08117	91.54422	1

Fragment Spectrum

100320_Total_17+18_ChymoTargeted.raw #14038 RT: 90.5494 min
ITMS, 712.3749@hcd30.00, z=+2, Mono m/z=712.37494 Da, MH+=1423.74260 Da, Match Tol.=0.6 Da

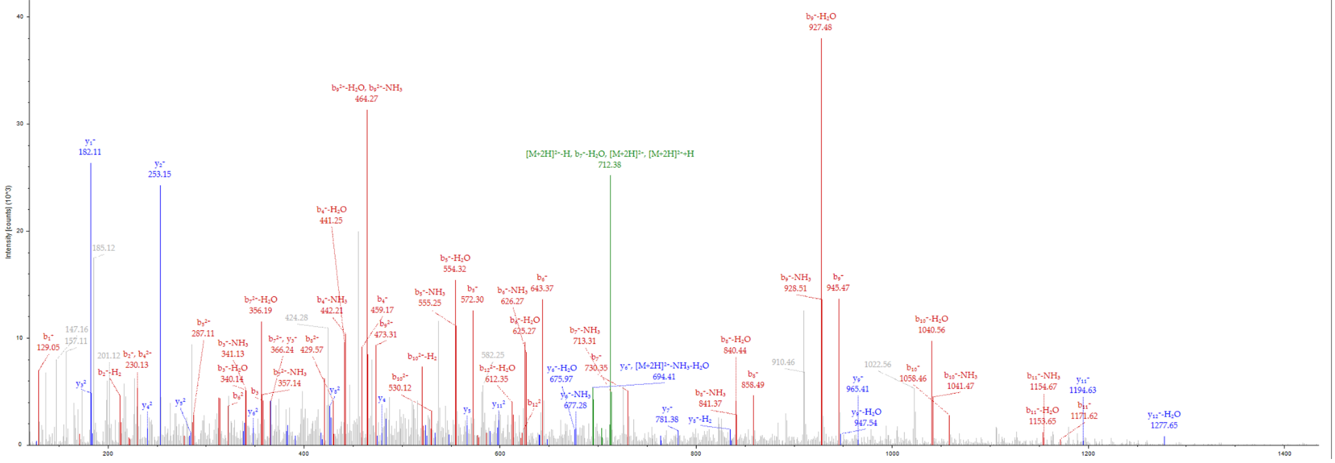

Annotated ITMS2 spectra for PSM corresponding to peptide QTQTIASQIIAY which is unique to the Bristol deletion variant of the S glycoprotein.
